# Gastrointestinal protozoa in pet cats from Anhui province: prevalence and molecular characterization

**DOI:** 10.3389/fcimb.2025.1522176

**Published:** 2025-01-27

**Authors:** Hao Zhang, Xing Tong, Zhonghui Ma, Tao Zhang, Feng Wu, Qiong Wu, Congshan Yang, Chunyang Han

**Affiliations:** ^1^ College of Veterinary Medicine, Anhui Agricultural University, Hefei, Anhui, China; ^2^ Chongxin’an Animal Hospital, Hefei, Anhui, China; ^3^ Jiujiang District Animal Husbandry and Veterinary Service Center, Wuhu, China

**Keywords:** gastrointestinal protozoa, pet cats, prevalence, zoonotic, public health

## Abstract

**Introduction:**

To investigate the prevalence of *Tritrichomonas foetus*, *Pentatrichomonas hominis*, *Giardia intestinalis*, *Cryptosporidium*, *Microsporidium*, and *Sarcocystis* in domestic cats in Anhui Province, China, and their potential role as zoonotic hosts for human infection, a total of 304 fecal samples from two different sources were screened for the presence of related pathogens.

**Methods:**

Using microscopy, along with polymerase chain reaction (PCR) or nested PCR amplification, followed by genotyping through sequence analysis.

**Results:**

The infection rates of *T. foetus*, *P. hominis*, *G. intestinalis*, *Cryptosporidium*, *Enterocytozoon bieneusi*, and *Sarcocystis* were 5.6%, 0%, 1.7%, 0.7%, 2.6%, and 0%, respectively. The evolutionary relationships and genetic characteristics of *G. intestinalis* based on the GDH gene, *Cryptosporidium* based on the SSU rRNA gene, and *E. bieneusi* based on the ITS sequence were assessed: five cases of *G. intestinalis* were identified, with four belonging to assemblage F and one to zoonotic assemblage B, two *Cryptosporidium* cases were identified as *Cryptosporidium felis*, and all eight *E. bieneusi* cases were identified as belonging to group 1, with three cases being genotype D, three EbpA, and two EbpC.

**Discussion:**

Age, neutering status, and deworming were identified as potential risk factors. Further analysis revealed that diarrhea, as a clinical symptom, could serve as an indicator for pathogen infection. Although the pathogen infection rates detected in this study were relatively low, their zoonotic transmission potential cannot be ignored. Therefore, special attention should be paid, and it is essential to establish targeted prevention plans.

## 1 Introduction

The health of pets has becoming a significant concern as the pet industry continues to grow. Among companion animals, cats are particularly vulnerable to gastrointestinal parasitic infections, which threaten their health and pose zoonotic risks to humans (Morelli et al., 2021). Common parasites include trichomonads, *Giardia intestinalis*, *Cryptosporidium*, *Microsporidium*, and *Sarcocystis* (Lappin, 2005). These parasites are distributed globally and can cause chronic diarrhea and other gastrointestinal symptoms, mainly through the ingestion of contaminated food or water (Ryu et al., 2007; Rosypal et al., 2012; Li et al., 2019b; Itoh et al., 2020; Strazdaitė-Žielienė et al., 2022).

Trichomonads, such as *Tritrichomonas foetus* and *Pentatrichomonas hominis*, are important pathogens causing chronic diarrhea in cats, especially in the young or immunocompromised individuals (Levy et al., 2003). Both species have been reported to infect humans, emphasizing their zoonotic potential (Maritz et al., 2014). Similarly, *G. intestinalis* exhibits host specificities, predominantly infecting cats through assemblage F. Zoonotic assemblages A and B have also been identified (Heyworth, 2016). Among *Cryptosporidium* species in cats, host-specific strains like *Cryptosporidium felis* coexist with zoonotic species such as *Cryptosporidium parvum* and *Cryptosporidium hominis* (Önder et al., 2021). These species often cause self-limiting diarrhea; however, transmission from companion animals can lead to secondary infections, which may become severe or life-threatening, particularly in immunocompromised individuals (Li et al., 2021; Jiang et al., 2023). *Microsporidium* species, particularly *Enterocytozoon bieneusi*, are increasingly recognized for their zoonotic importance (Santín and Fayer, 2011) and are considered among the most frequently diagnosed species in human infections (Karim et al., 2014). Group 1 genotypes often infect both humans and cats, while other groups exhibit higher host specificity (Xu et al., 2016). Although *Sarcocystis* infections in cats are less frequently reported, cats act as definitive hosts for various species, underscoring their role in potential disease transmission (Kirkpatrick and Dubey, 1987).

Although many global studies have examined feline parasitic infections, systematic epidemiological data specific to China remain scarce, particularly regarding their zoonotic potential. This study focuses on assessing the prevalence of gastrointestinal parasites in cat fecal samples from 12 cities in Anhui Province. The results aim to enhance understanding of the epidemiology of these infections and their risks to human health, providing valuable insights to inform public health measures and reduce the likelihood of zoonotic transmission.

## 2 Materials and methods

### 2.1 Ethical approval

This study adhered to the Guide for the Care and Use of Laboratory Animals set forth by the Ministry of Health, China. The protocol received approval from the Research Ethics Committee of the Anhui Agricultural University (number AHAUB2022020). All cat owners participating in this study were informed and consented to the examination. Throughout the sample collection process, measures were taken to minimize any stress or stimulation experienced by the animals.

### 2.2 Sample collection and microscopic examination

**Figure 1:**
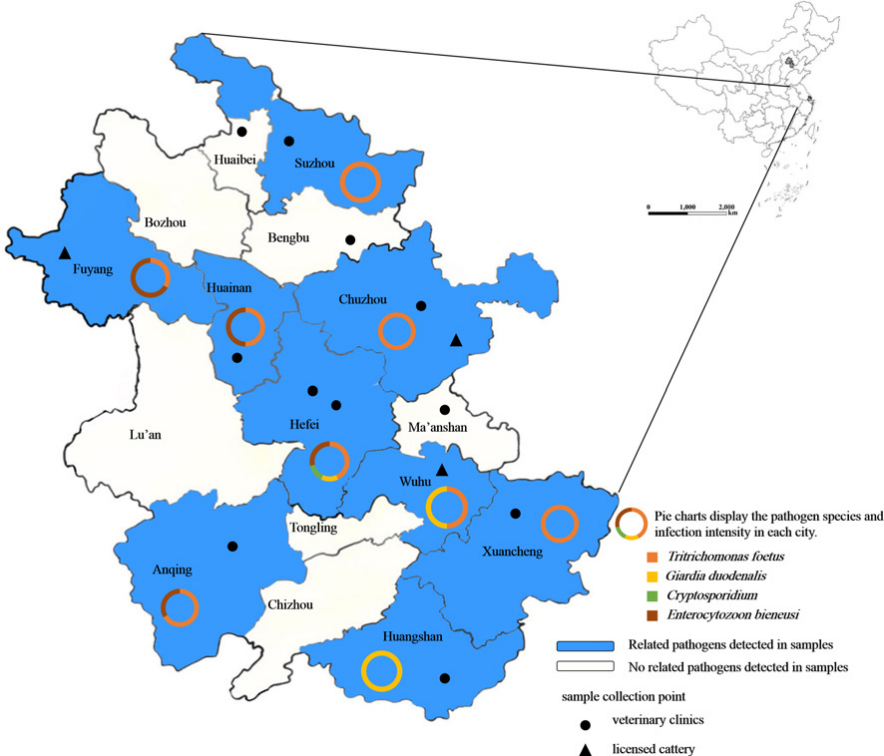
Geographical distribution of fecal samples used for detecting intestinal protozoa in pet cats in Anhui Province, China. The shaded areas indicate regions where related pathogens were detected, and the legend shows the locations and types of sampling sites.

During from July to December 2023, fecal samples from 304 pet cats (**Table 1**) were collected from 11 veterinary clinics and 3 licensed catteries in 12 cities of Anhui Province, China (**Figure 1**). The samples were stored in ice boxes and transported to the Veterinary Parasite Laboratory, College of Animal Science and Technology at Anhui Agricultural University in Hefei. All feces samples were kept at 4°C until processing within one week. The sample information included gender, age, neuter status (analyzed among individuals older than 6 months), deworming status, and health conditions (diarrhea, weight loss, vomiting and anorexia).

**Table 1:** Cities and the number of collected cat fecal samples in Anhui Province, China, along with positive samples and corresponding infection rates for each city.

Regions	No. Samples	Positive samples and infection rates					
		*T. foetus*	*P. hominis*	*G. intestinalis*	*C. felis*	*E. bieneusi*	*Sarcocystis*
Anqing	24	2 (8.3%)	0	0	0	1 (4.2%)	0
Bengbu	20	0	0	0	0	0	0
Chuzhou	35	3 (8.6%)	0	0	0	0	0
Fuyang	25	1 (4.0%)	0	0	0	2 (8.0%)	0
Hefei	50	6 (12.0%)	0	2(4.0%)	2 (4.0%)	4 (8.0%)	0
Huangshan	26	0	0	2 (7.7%)	0	0	0
Huaibei	21	0	0	0	0	0	0
Huainan	23	1 (4.3%)	0	0	0	1 (4.3%)	0
Ma’ansahn	20	0	0	0	0	0	0
Suzhou	19	2 (10.5%)	0	0	0	0	0
Wuhu	21	1 (4.8%)	0	1 (4.8%)	0	0	0
Xuancheng	20	1 (5.0%)	0	0	0	0	0
Total	304	17 (5.6%)	0	5 (1.6%)	2 (0.7%)	8 (2.6%)	0

A small amount of feces was directly smeared onto a slide, mixed with a few drops of phosphate-buffered saline (PBS) using a clean toothpick to ensure even dispersion, and then covered with a cover slip. Subsequently, the smears were examined under a microscope using bright-field microscopy at magnifications of 100× and 400×.

### 2.3 DNA extraction and PCR detection

According to the manufacturer’s instructions, genomic DNA was extracted from approximately 200 mg of fecal samples using the TIANamp Stool DNA Kit (TIANGEN Biotech Co., Ltd., Beijing, China) and stored at -20°C for subsequent PCR detection.

Specific target genes of *T. foetus*, *P. hominis*, *G. intestinalis*, *Cryptosporidium*, *E. bieneusi*, and *Sarcocystis* were amplified to determine the presence of pathogenic infections through PCR or nested PCR. The primer sequences, product lengths, and annealing temperatures are listed in **Table 2**. After amplification, the PCR products were validated by agarose gel electrophoresis.

**Table 2:** Primer names and sequences for the detection of *T. foetus* and other protozoan molecular identification, along with PCR cycling conditions.

Species	Target gene	Primer	Sequence (5’-3’)	Cycling conditions	Size(bp)	Ref.
*T. foetus*	5.8S rRNA and ITS1/ITS2	TFR3	CGGGTCTTCCTATATGAGACAGAACC	25s/94°C, 25s/56°C, 60s/72°C, 34cycles	347	(Felleisen et al., 1998)
		TFR4	CCTGCCGTTGGATCAGTTTCGTTAA			
*P. hominis*	18S rRNA	Th3	TGTAAACGATGCCGACAGAG	60s/95°C, 60s/64°C, 2min/72°C, 35cycles	339	(Gookin et al., 2007)
		Th5	CAACACTGAAGCCAATGCGAG			
*G. intestinalis*	GDH	GDH1	ATCTTCGAGAGGATGCTTGAG	25s/94°C, 25s/58°C, 60s/72°C, 35cycles	778	(Homan et al., 1998.)
		GDH4	AGTACGCGACGCTGGGATACT			
*Cryptosporidium*	18S rRNA	cry18SF1	TTCTAGAGCTAATACATGCG	PCR1,PCR2: 45s/94°C, 45s/55°C, 60s/72°C, 35cycles	820	(Xiao et al., 2004)
		cry18SR1	CCCATTTCCTTCGAAACAGGA			
		cry18SF2	GGAAGGGTTGTATTATTAGATAAAG			
		cry18SR2	AAGGAGTAAGGAACAACCTCCA			
*E. bieneusi*	ITS	EBITS3	GGTCATAGGGATGAAGAG	PCR1:30s/94°C, 30s/57°C, 40s/72°C, 34cycles PCR2:30s/94°C, 30s/55°C, 40s/72°C, 34cycles	392	(Karim et al., 2015)
		EBITS4	TTCGAGTTCTTTCGCGCTC			
		EBITS1	GCTCTGAATATCTATGGCT			
		EBITS2.4	ATCGCCGACGGATCCAAGTG			
*Sarcocystis*	18S rRNA	1L	CCATGCATGTCTAAGTATAAGC	PCR1,PCR2: 40s/94°C, 30s/50°C, 60s/72°C, 35cycles	960	(Lau et al., 2013)
		1H	TATCCCCATCACGATGCATAC			
		3L	CTAGTGATTGGAATGATGGG			
		2H	ACCTGTTATTGCCTCAAACTTC			

### 2.4 Cloning construction and sequencing

PCR-positive products were purified using the FastPure Gel DNA Extraction Mini Kit (Vazyme Biotech Co., Ltd., Nanjing, China), following the manufacturer’s instructions. The purified products were cloned into the pCE2 TA/Blunt-Zero vector from the 5 min TA/Blunt-Zero Cloning Kit (Vazyme Biotech Co., Ltd., Nanjing, China). The recombinant vectors were then transformed into *Escherichia coli* DH5α competent cells (Tolobio, Shanghai, China). Selective cloning was performed on Luria-Bertani (LB) agar plates containing ampicillin resistance. Positive clones were identified by PCR, and at least three positive clones corresponding to each PCR product were sequenced to confirm the sequence (Tsingke, Nanjing, China).

### 2.5 Sequence and phylogenetic analyses

All positive sequences obtained by sequencing underwent molecular analysis and genetic identification to determine species. Nucleotide sequences were assembled and edited using the Lasergene program (DNASTAR, Inc, Madison, WI, USA). BLAST analysis was performed on the nucleotide sequences in the GenBank database. Phylogenetic relationships among species were evaluated using the Neighbor-Joining (NJ) method in MEGA 11 (https://www.megasoftware.net/), with branch reliability assessed by 1000 bootstrap replicates. Phylogenetic trees representing the evolutionary relationships of the species were subsequently constructed.

The obtained sequences were submitted to GenBank under the following accession numbers: *T. foetus* rRNA/ITS sequences: PP999319–PP999335, *G. intestinalis* GDH sequences: PQ084557–PQ084561, *Cryptosporidium* 18S rRNA sequences: PQ008240–PQ008241, *E. bieneusi* 18S rRNA sequences: PQ008242–PQ008246, PQ411259-PQ411261.

### 2.6 Statistical analysis

Statistical analyses were conducted using GraphPad Prism version 9.5 (GraphPad Software, San Diego, CA, USA). Fisher’s exact test was employed to compare infection rates across various categorical variables, including age, sex, neuter status, deworming history, and clinical symptoms (such as diarrhea, weight loss, vomiting and anorexia). The association between these factors and gastrointestinal parasite infection was further assessed by calculating the odds ratio (OR) with a 95% confidence interval (CI), which provided an estimate of the relative risk of infection for each factor. Statistical significance was set at p < 0.05 for all tests.

All statistical analyses were two-tailed, and care was taken to address potential biases due to small sample sizes or uneven distributions across groups. This method ensured that the identified risk factors were valid and offered meaningful insights into the relationship between these factors and pathogen infection.

## 3 Results

### 3.1 Prevalence of gastrointestinal protozoan infections – microscopy and PCR results

All collected fecal samples (n=304) were examined using both microscopy and PCR techniques. Microscopy identified 12 cases of *Giardia*-like parasites. Based on previously recorded clinical symptoms, all 12 cases presented with recurrent diarrhea. According to the PCR results, 17 cases of *T. foetus* (5.6%), 5 cases of *G. intestinalis* (1.6%), 2 cases of *Cryptosporidium* (0.7%), and 8 cases of *E. bieneusi* (2.6%) were identified, while *P. hominis* and *Sarcocystis* were not detected (**Table 1**). Among these, there were two cases of mixed infection with *T. foetus* and *G. intestinalis*, originating from Hefei and Wuhu, respectively. The detection rate of *T. foetus* was highest in Hefei (12%, 6/50; p=0.04), and the highest detection rate of *G. intestinalis* was found in Huangshan (7.7%, 2/26; p=0.06). The detection rates of *E. bieneusi* were highest in Fuyang (8%, 2/25; p=0.13) and Hefei (8%, 4/50; p=0.03), while the only two cases of *Cryptosporidium* were detected in Hefei.

The infection rates of pathogens in cats of different ages, sexes, neutering status, and deworming conditions are shown in **Table 3**. The detection rate varied by age. According to the age group data, the detection rate in cats under 6 months (<6 months: 20.7%, 12/58; p=0.005) was significantly higher than in those aged 6-12 months (10.6%, 13/123; p=0.84) and over 12 months (>12 months: 4.1%, 5/123; p=0.16). The data for gender groups did not show a significant difference (p>0.05). The data for neutering status showed differences, with a higher detection rate in the non-neutered group (14.1%, 11/89; p=0.04). Similarly, there were differences in deworming conditions, with the detection rate in the untreated group (30.8%, 16/52) being significantly higher than in the treated group (5.6%, 14/252) (p<0.0001).

**Table 3:** Infection of the pathogen in different cats.

Variable	No. Samples	Gastrointestinal protozoa		
		No. Positive (%)	OR (95%CI)	p-value
Age				
<6 months	58	12 (20.7%)	3.30 (1.5-7.4)	0.005**
6-12 months	123	13 (10.6%)	1.14 (0.5-2.4)	0.84
≥12 months	123	5 (4.1%)	0.45 (0.2-1.2)	0.16
Gender				
Male	155	17 (11.0%)	1.29 (0.6-2.7)	0.57
Female	149	13 (8.7%)	0.78 (0.4-1.6)	
Neutered				
Yes	157	7 (4.7%)	0.33 (0.1-0.9)	0.04*
No	89	11 (14.1%)	3.02 (1.1-7.8)	
Deworming				
Yes	252	14 (5.6%)	0.13 (0.1-0.3)	<0.0001****
No	52	16 (30.8%)	7.56 (3.3-16.5)	

When p<0.05 and OR>1, potential risk factors for infection are considered to exist.

* indicates statistical significance; ** indicates high statistical significance; **** indicates very high statistical significance.

Moreover, the odds ratio data (**Table 3**) showed that cats under 6 months of age (<6 months: OR=3.30, p=0.005) were more susceptible to pathogen infection, indicating the susceptibility of cats in this age group. Non-neutered cats (OR=3.02, p=0.04) also had a significantly higher susceptibility to infection compared to other cats. The untreated group (OR=7.56, p<0.0001) also demonstrated a significant risk of pathogen transmission among domestic cats.

### 3.2 Association between gastrointestinal parasites and clinical symptoms

Among the 304 collected samples, there were 62 cats (**Table 4**) exhibiting clinical symptoms, with 30 cases (48.4%) testing positive for pathogens, including two cases of mixed infection. Odds ratio data analysis was conducted to determine the risk factors associated with the transmission of gastrointestinal protozoa. Specifically, diarrhea (60.6%, 20/33; OR=2.92, p=0.047) may be a significant risk factor for infection and can also serve as an important indicator of gastrointestinal parasite infection.

**Table 4:** Association between gastrointestinal parasites and clinical symptoms.

Clinical symptom	No. Samples	Gastrointestinal protozoa		
		No. Positive (%)	OR (95%CI)	p-value
diarrhea	33	20 (60.6%)	2.92 (1.0-8.4)	0.047*
weight loss	8	3 (37.5%)	0.60 (0.1-2.7)	0.71
vomiting	13	5 (38.6%)	0.60 (0.2-2.0)	0.54
anorexia	8	2 (25.0%)	0.31 (0.1-1.4)	0.26
Total	62	30 (48.4%)		

When p<0.05 and OR>1, potential risk factors for infection are considered to exist.

* indicates statistical significance.

### 3.3 *Cryptosporidium* species in cats

**Figure 2:**
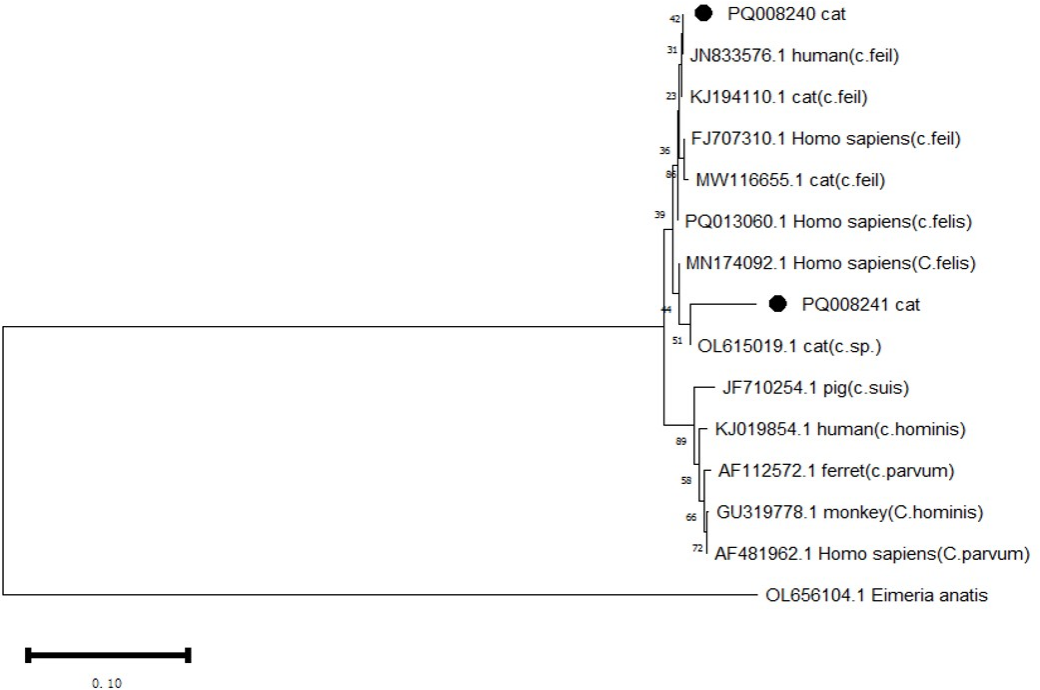
Phylogenetic analysis of the 18s rRNA gene was performed using MEGA 11 software, revealing the phylogenetic relationships of the *Cryptosporidium* species isolated in this study with other known species of the genus *Cryptosporidium*. *Eimeria anatis* (OL656104.1) was used as the root. The tree was constructed using the NJ method based on the Maximum Composite Likelihood (MCL) model. The *Cryptosporidium* species discovered in this study are marked with •.

DNA sequencing and sequence analysis of the 18s rRNA PCR products from two *Cryptosporidium*-positive samples revealed that both belong to *C. felis* (**Table 5**, **Figure 2**). The nucleotide sequence with GenBank accession number PQ008240 showed 100% identity to the sequence with accession number JN833576. The positive isolate was detected in Shanghai. Notably, isolates with over 95% identity have records of human hosts. The nucleotide sequence with accession number PQ008241 exhibited 96.6% identity to the sequence with accession number OL615019 (Köseoğlu et al., 2022). This isolate was obtained from the feces of a stray cat in Izmir, Turkey, and based on phylogenetic tree analysis, the sample shows significant divergence.

**Table 5:** Species of *Cryptosporidium*, genotypes of *E. bieneusi*, and assemblages of *G. intestinalis* isolates determined by PCR and sequence analysis in each positive animal.

City	Source^a^	Age^b^	Gender	*G. intestinalis*	*Cryptosporidium*	*E. bieneusi*
Hefei	VC	2 M	Female		*C. felis*	
Hefei	VC	8 M	Male		*C. felis*	
Hefei	VC	18 M	Male	Assemblage: F		
Hefei	VC	3 M	Male	Assemblage: F		
Wuhu	LC	11 M	Male	Assemblage:B		
Huangshan	VC	5 M	Female	Assemblage: F		
Huangshan	VC	8 M	Female	Assemblage: F		
Fuyang	LC	7 M	Female			EbpA
Hefei	VC	9 M	Female			EbpC
Hefei	VC	5 M	Female			D
Huainan	VC	15 M	Female			D
Hefei	VC	4 M	Male			D
Hefei	VC	7 M	Male			EbpC
Bengbu	VC	10 M	Male			EbpA
Fuyang	LC	4 M	Male			EbpA

a VC, veterinary clinic; LC, licensed cattery.

b M, month.

### 3.4 Genotypes and phylogeny of *E. bieneusi* in cats

DNA sequencing and sequence analysis of the ITS PCR products from 8 *E. bieneusi* positive samples revealed the presence of three known genotypes: D, EbpA, and EbpC (**Table 5**). Among them, EbpA is considered to be associated with genotype D. The genotypes D and EbpC differ in the ITS sequence by 1 to 6 single nucleotide polymorphisms (SNPs). The number of detected cases for genotype D and EbpA is the same (n=3), while EbpC has a detection count of (n=2).

**Figure 3:**
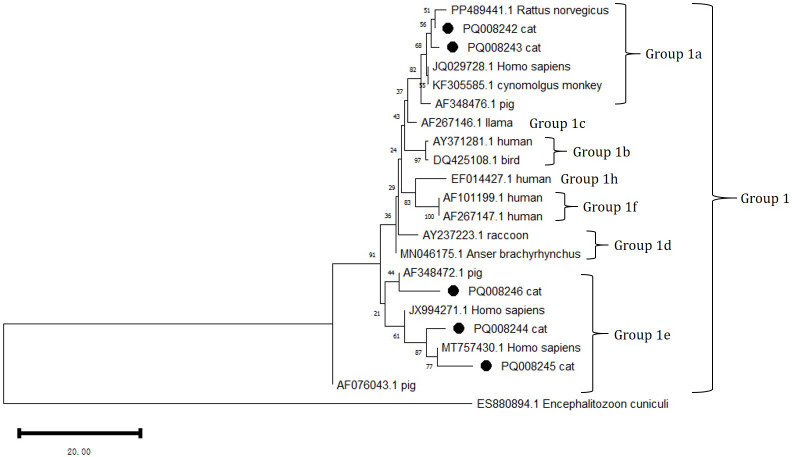
Phylogenetic analysis of the ITS rRNA gene using MEGA 11 software revealed the phylogenetic relationship of the *E. bieneusi* genotypes isolated in this study with other known zoonotic pathogens in the first group of *E. bieneusi*. The ITS tree is rooted with ES880894.1 *Encephalitozoon cuniculi*. In this analysis, the nucleotide sequences with GenBank accession numbers PQ088244 = PQ411259, PQ088246 = PQ411261, and PQ008242 = PQ44260 were included. The grouping of subgroup 1 was primarily referenced from Lin Wang and Lihua Xiao, 2013. The phylogenetic tree was constructed using the NJ method based on the MCL model. The *E. bieneusi* genotypes identified in this study are marked with •.

Phylogenetic analysis of all *E. bieneusi* ITS genotypes identified in this study shows that genotypes D (subgroup 1a), EbpC (1e), and EbpA (1e) are classified into the first group of zoonotic pathogens (**Figure 3**).

### 3.5 Genetic assemblages of *G. intestinalis* in cats

**Figure 4:**
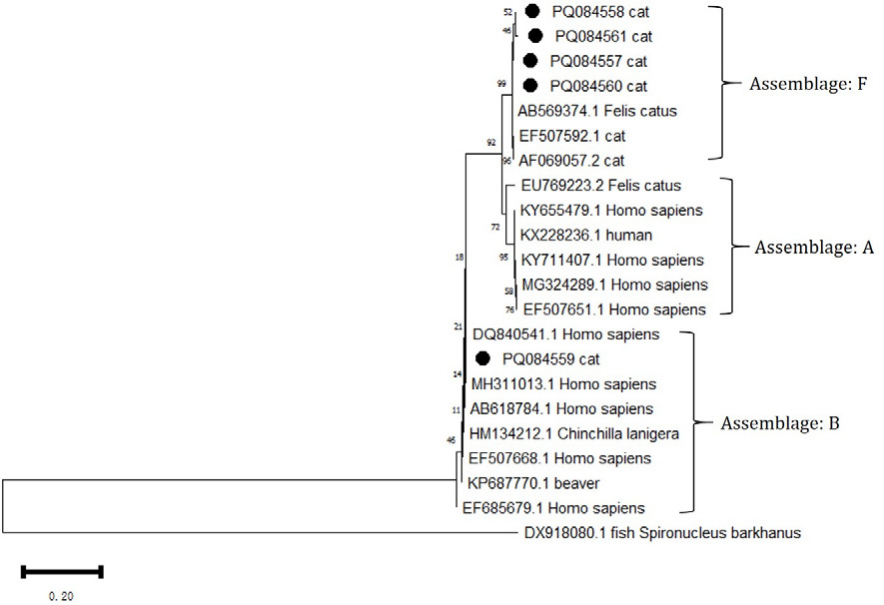
Phylogenetic analysis of the GDH gene was performed using MEGA 11 software, revealing the phylogenetic relationships of the *G. intestinalis* genotypes isolated in this study with other known species of the genus *Giardia*. The evolutionary tree is rooted with DX918080.1 *Spironuclenus barkhanus*. The tree encompasses Assemblages A, B, and F. The phylogenetic tree was constructed using the NJ method based on the MCL model. The *G. intestinalis* genotypes identified in this study are marked with •.

All five positive *G. intestinalis* PCR products were successfully sequenced at the GDH locus. Through BLAST analysis, comparisons were made with sequences in the GenBank database, identifying that *G. intestinalis* belongs to Assemblages B and F (**Figure 4**). Among them, Assemblage B is a zoonotic genetic composition, while Assemblage F exhibits notable host specificity. The sources, ages, and genders of the positive animals are shown in **Table 5**.

## 4 Discussion

This is the first report describing the prevalence and genetic characteristics of gastrointestinal parasites in cats from Anhui Province, China, primarily detecting six pathogens: *T. foetus*, *P. hominis*, *G. intestinalis*, *Cryptosporidium*, *E. bieneusi*, and *Sarcocystis*. Apart from *P. hominis* and *Sarcocystis*, the remaining pathogens were all detected. The prevalence of infection in the cats investigated in Anhui Province in this study was 9.9% (30/304), with two cases of mixed infections involving *T. foetus* and *G. intestinalis*.

*T. foetus* is the most common parasite causing diarrhea in cats (Xenoulis et al., 2013), with reports from various countries and regions around the world (Yao and Köster, 2015). However, epidemiological studies of *T. foetus* in China are limited (Köster et al., 2015). In this study, PCR detection of its conserved sequence revealed a detection rate of 5.6% (Felleisen et al., 1998), making it the most common parasite observed in this study, a rate similar to that observed in Japan (8.8%) (Doi et al., 2012). Notably, there were two cases of mixed infection with *G. intestinalis*, both cats being under 1 year of age and having a history of diarrhea. In this study, *P. hominis* was not detected. Cases of *P. hominis* infection in cats are indeed rare (Romatowski, 2000; Bouzid et al., 2015), but its potential risk as a zoonotic disease cannot be ignored.

*G. intestinalis* infections in cats have been reported in many countries and regions, including Heilongjiang, Henan, Shanghai, and Guangdong in China (Bouzid et al., 2015; Li et al., 2015; Xu et al., 2016; Pan et al., 2018; Li et al., 2024). In this study, the infection rate was 1.6%. Sequence analysis identified one isolate as assemblage B, and the other four as assemblage F. Consistently, assemblage F is the most common genotype found in cats (Feng and Xiao, 2011). As *G. intestinalis* is recognized as a zoonotic pathogen, its zoonotic potential and the contribution of animal sources to the human disease burden are particularly important. One isolate in this study belonged to assemblage B, which aligns with reports of human infections and may represent a novel subtype within sub-assemblage BIV (Abe and Teramoto, 2012). At the same time, although assemblage F exhibits host specificity to cats, there is still a risk of human infection in areas with poor sanitary conditions (Pipiková et al., 2020).

Global epidemiological surveys on *Cryptosporidium* are numerous, with infection rates ranging from 0.6% to 40.8% (Meng et al., 2021). In China, detailed reports on the prevalence and genetic characteristics of *Cryptosporidium* in cats are only available for Heilongjiang, Henan, Shanghai, and Guangdong (Li et al., 2015; Xu et al., 2016; Li et al., 2019a, 2024). In this study, the infection rate of *Cryptosporidium* in cats was 0.7%, with both cases detected in Hefei. Previous reports also indicated an infection rate of 3.3% in cats in Hefei (Li et al., 2019c), which is similar to the detection rate of 4% in Hefei cats in this study. The isolates in this study were identified as *C. felis*, the most common species found in cats, but in some developing countries, there are widespread cases of human infections (Xiao, 2010).

Microsporidiosis in cats is an emerging zoonotic public health issue that has gained some attention globally in recent years (Meng et al., 2021). In this study, the infection rate of this pathogen was 2.6%, and phylogenetic analysis showed that all eight positive isolates belonged to zoonotic Assemblage 1. In China, the transmission of this pathogen is highly prevalent in water sources (especially wastewater), providing an important medium for the survival and spread of spores, whether through direct drinking water or indirectly through irrigation, food washing, or bathing water (Qiu et al., 2019). Therefore, the management of water sources and sanitation measures in daily life are particularly important. The global burden of microsporidiosis in cats is significant, and the lack of sufficient research in developing countries indicates that greater attention should be given to the prevalence of microsporidiosis in cats.

Reports of *Sarcocystis* in cat feces are rare. A retrospective analysis of related infections in North America found an infection rate of only 0.1% (Nagamori et al., 2020). In this study, no cases of *Sarcocystis* infection in cats’ feces were detected. *Sarcocystosis* infections exhibit complete enzootic stability in most parts of the world (Rommel, 1985). Domestic cats serve as definitive hosts for several *Sarcocystosis* species, such as *Sarcocystis felis*, and wild felids are also commonly infected (Cañón-Franco et al., 2016). Although cats are not as frequently associated with human infections as other definitive hosts, their role in the zoonotic *Sarcocystis* species life cycle should not be overlooked (Fayer et al., 2015).

Meanwhile, we analyzed some related risk factors for infection, and statistical analysis revealed that age, neutering status, and deworming were potential risk factors with statistically significant differences, which align with expectations. Young hosts are more susceptible to infections due to weakened immunity, increasing their infection risks. In this study, there were four cases from veterinary clinics involving rescued stray kittens all exhibited clinical symptoms of emaciation and parasitic infections, highlighting the higher risk faced by young cats in harsh environments. Neutering, as the primary intervention for most pet cats (Barrios et al., 2023), has previously been identified as a potential risk factor for infection (Colella et al., 2022), possibly due to reduced external exposure behavior in neutered cats (Mohamed et al., 2013). However, due to the small sample size and lack of behavioral data, we cannot conclusively determine whether neutering directly leads to a higher or lower infection rate. Interestingly, our three cases all come from catteries, all of which provided fecal samples testing positive for pathogen infections. Housing conditions and management practices in catteries may increase the risk of infection to some extent (Gookin et al., 2004). Gastrointestinal parasitic infections in cats are often accompanied by clinical symptoms such as diarrhea, emaciation, vomiting, and anorexia (Abbas et al., 2022). In this study, we found that diarrhea frequently indicated the potential presence of parasitic infection. Some samples exhibited extreme emaciation, which drew our attention, and subsequent tests confirmed parasitic infections. Although statistical analysis did not show significant differences, the fact that parasites extract nutrients from the host’s gastrointestinal tract to sustain their own life activities, leading emaciation (Xenoulis et al., 2013), suggests that emaciation may also indicate a possible infection.

The value of cats as pets has been extensively studied, and their interactions with humans have gained significant attention (Turner, 2017; Hart et al., 2018). Therefore, we cannot ignore their potential role as intermediate hosts for the transmission of parasites. However, this study only involved collecting fecal samples from pet cats, and no samples were collected from the owners. Consequently, there is a lack of molecular evidence to assess the potential transmission risk between pet cats and humans. Unfortunately, as the number of samples from stray cats was very limited, it was also difficult to make reliable comparisons regarding the risk factors in different environments.

This study also detected genetic combinations of pathogens that pose a risk of human infection, highlighting the importance of monitoring zoonotic transmission. Pet owners should establish targeted prevention plans, paying attention to outdoor exposure to reduce the likelihood of infection. Additionally, regular monitoring, eliminating exposure to infectious sources, and isolating sick animals remain effective methods for reducing the spread of pathogens.

## 5 Conclusion

This study identifies the prevalence of *T. foetus*, *P. hominis*, *G. intestinalis*, *Cryptosporidium*, *E. bieneusi*, and *Sarcocystis* in pet cats in Anhui Province, along with their phylogenetic characteristics. Although the prevalence is low, zoonotic combinations were detected. Key risk factors include young age, lack of neutering, and absence of deworming. Given the close contact between pet cats and humans, infection risks for cat owners may rise. Targeted management strategies are essential to reduce environmental contamination and safeguard both pet and public health. Further large-scale studies, including stray cats, are needed to evaluate the zoonotic risks of these pathogens.

## Data Availability

The original contributions presented in the study are publicly available. This data can be found at: https://www.ncbi.nlm.nih.gov/nuccore/; *T. foetus* rRNA/ITS sequences: PP999319–PP999335, *G. intestinalis* GDH sequences: PQ084557–PQ084561, *Cryptosporidium* 18S rRNA sequences: PQ008240–PQ008241, *E. bieneusi* 18S rRNA sequences: PQ008242–PQ008246, PQ411259–PQ411261.

## References

[B1] Abbas I. Al-Araby M. Elmishmishy B. El-Alfy E.-S. (2022). Gastrointestinal parasites of cats in Egypt: high prevalence high zoonotic risk. BMC Veterinary Res. 18, 420. doi: 10.1186/s12917-022-03520-0 PMC9706847 36447265

[B2] Abe N. Teramoto I. (2012). Molecular evidence for person-to-person transmission of a novel subtype in *Giardia duodenalis* assemblage B at the rehabilitation institution for developmentally disabled people. Parasitol. Res. 110, 1025–1028. doi: 10.1007/s00436-011-2564-4 21786066

[B3] Barrios F. Suárez G. Udell M. A.R. Damián J. P. (2023). Characterization of the domestic cat population of Uruguay: breeds, coat colors, hair length, lifestyle, sex and spay/neuter status according to guardian report. Animals 13, 1963. doi: 10.3390/ani13121963 37370473 PMC10295408

[B4] Bouzid M. Halai K. Jeffreys D. Hunter P. R. (2015). The prevalence of *Giardia* infection in dogs and cats, a systematic review and meta-analysis of prevalence studies from stool samples. Veterinary Parasitol. 207, 181–202. doi: 10.1016/j.vetpar.2014.12.011 25583357

[B5] Cañón-Franco W. A. López-Orozco N. Christoff A. U. De Castilho C. S. De Araújo F. A. Verma S. K.. (2016). Molecular and morphologic characterization of *Sarcocystis felis* (Apicomplexa: Sarcocystidae) in South American wild felids from Brazil. Veterinary Parasitol. 217, 15–20. doi: 10.1016/j.vetpar.2015.12.025 26827854

[B6] Colella V. Wongnak P. Tsai Y.-L. Nguyen V.-L. Tan D. Y. Tong K. B.Y.. (2022). Human social conditions predict the risk of exposure to zoonotic parasites in companion animals in East and Southeast Asia. Commun. Med. 2, 144. doi: 10.1038/s43856-022-00210-8 36380151 PMC9666534

[B7] Doi J. Hirota J. Morita A. Fukushima K. Kamijyo H. Ohta H.. (2012). Intestinal *Tritrichomonas suis* (=*T. foetus*) Infection in Japanese Cats. J. Veterinary Med. Sci. 74, 413–417. doi: 10.1292/jvms.11-0171 22104396

[B8] Fayer R. Esposito D. H. Dubey J. P. (2015). Human infections with *Sarcocystis* species. Clin. Microbiol. Rev. 28, 295–311. doi: 10.1128/CMR.00113-14 25715644 PMC4402950

[B9] Felleisen R. S.J. Lambelet N. Bachmann P. Nicolet J. Müller N. Gottstein B. (1998). Detection of *Tritrichomonas foetus* by PCR and DNA Enzyme Immunoassay Based on rRNA Gene Unit Sequences. J. Clin. Microbiol. 36, 513–519. doi: 10.1128/JCM.36.2.513-519.1998 9466768 PMC104569

[B10] Feng Y. Xiao L. (2011). Zoonotic potential and molecular epidemiology of. Giardia Species Giardiasis. Clin. Microbiol. Rev. 24, 110–140. doi: 10.1128/CMR.00033-10 21233509 PMC3021202

[B11] Gookin J. L. Stauffer S. H. Levy M. G. (2007). Identification of *Pentatrichomonas hominis* in feline fecal samples by polymerase chain reaction assay. Veterinary Parasitol. 145, 11–15. doi: 10.1016/j.vetpar.2006.10.020 17127004

[B12] Gookin J. L. Stebbins M. E. Hunt E. Burlone K. Fulton M. Hochel R.. (2004). Prevalence of and risk factors for feline *Tritrichomonas foetus* and *Giardia* infection. J. Clin. Microbiol. 42, 2707–2710. doi: 10.1128/JCM.42.6.2707-2710.2004 15184456 PMC427826

[B13] Hart L. A. Hart B. L. Thigpen A. P. Willits N. H. Lyons L. A. Hundenski S. (2018). Compatibility of cats with children in the family. Front. Veterinary Sci. 5. doi: 10.3389/fvets.2018.00278 PMC6252378 30510933

[B14] Heyworth M. F. (2016). *Giardia duodenalis* genetic assemblages and hosts. Parasite 23, 13. doi: 10.1051/parasite/2016013 26984116 PMC4794627

[B15] Homan W. L. Gilsing M. Limper L. van Knapen F. (1998). Characterization of *Giardia duodenalis* by polymerase-chain-reaction fingerprinting. Parasitol. Res. 84, 707–714. doi: 10.1007/s004360050474 9766898

[B16] Itoh N. Iijima Y. Ogura I. Yonekura N. Kameshima S. Kimura Y. (2020). Molecular prevalence of trichomonad species from pet shop puppies and kittens in Japan. Rev. Bras. Parasitologia Veterinária 29, e014820. doi: 10.1590/s1984-29612020098 33237191

[B17] Jiang Y. Liu L. Yuan Z. Liu A. Cao J. Shen Y. (2023). Molecular identification and genetic characteristics of *Cryptosporidium* spp., *Giardia duodenalis*, and *Enterocytozoon bieneusi* in human immunodeficiency virus/acquired immunodeficiency syndrome patients in Shanghai, China. Parasites Vectors 16, 53. doi: 10.1186/s13071-023-05666-8 36739387 PMC9899406

[B18] Karim Md R. Dong H. Li T. Yu F. Li D. Zhang L.. (2015). Predomination and new genotypes of *Enterocytozoon bieneusi* in captive nonhuman primates in zoos in China: High genetic diversity and zoonotic significance. PloS One 10, e0117991. doi: 10.1371/journal.pone.0117991 25705879 PMC4338232

[B19] Karim Md R. Dong H. Yu F. Jian F. Zhang L. Wang R.. (2014). Genetic diversity in *Enterocytozoon bieneusi* isolates from dogs and cats in China: Host specificity and public health implications. J. Clin. Microbiol. 52, 3297–3302. doi: 10.1128/JCM.01352-14 24989604 PMC4313153

[B20] Kirkpatrick C. E. Dubey J. P. (1987). Enteric coccidial infections. Veterinary Clinics North America: Small Anim. Pract. 17, 1405–1420. doi: 10.1016/S0195-5616(87)50009-2 3127978

[B21] Köseoğlu A. E. Can H. Karakavuk M. Güvendi M. Değirmenci Döşkaya A. Manyatsi P. B.. (2022). Molecular prevalence and subtyping of *Cryptosporidium* spp. in fecal samples collected from stray cats in İzmir, Turkey. BMC Veterinary Res. 18, 89. doi: 10.1186/s12917-022-03190-y PMC8898748 35255909

[B22] Köster L. S. Chow C. Yao C. (2015). Trichomonosis in cats with diarrhoea in Hong Kong, China, between 2009 and 2014. J. Feline Med. Surg. Open Rep. 1, 2055116915623561. doi: 10.1177/2055116915623561 PMC5361988 28491403

[B23] Lappin M. R. (2005). Enteric protozoal diseases. Veterinary Clinics North America: Small Anim. Pract. 35, 81–88. doi: 10.1016/j.cvsm.2004.08.004 15627628

[B24] Lau Y. L. Chang P. Y. Subramaniam V. Ng Y. H. Mahmud R. Ahmad A. F.. (2013). Genetic assemblage of *Sarcocystis* spp. in Malaysian snakes. Parasites Vectors 6, 257. doi: 10.1186/1756-3305-6-257 24010903 PMC3847168

[B25] Levy M. G. Gookin J. L. Poore M. Birkenheuer A. J. Dykstra M. J. Litaker R.W. (2003). *Tritrichomonas foetus* and not *pentatrichomonas hominis* is the etiologic agent of feline trichomonal diarrhea. J. Parasitol. 89, 99–104. doi: 10.1645/0022-3395(2003)089[0099:TFANPH]2.0.CO;2 12659310

[B26] Li J. Dan X. Zhu K. Li N. Guo Y. Zheng Z.. (2019a). Genetic characterization of *Cryptosporidium* spp. and *Giardia duodenalis* in dogs and cats in Guangdong, China. Parasites Vectors 12, 571. doi: 10.1186/s13071-019-3822-z 31783765 PMC6884805

[B27] Li W. Feng Y. Santin M. (2019b). Host specificity of *Enterocytozoon bieneusi* and public health implications. Trends Parasitol. 35, 436–451. doi: 10.1016/j.pt.2019.04.004 31076351

[B28] Li W. Li Y. Song M. Lu Y. Yang J. Tao W.. (2015). Prevalence and genetic characteristics of *Cryptosporidium*, *Enterocytozoon bieneusi* and *Giardia duodenalis* in cats and dogs in Heilongjiang province, China. Veterinary Parasitol. 208, 125–134. doi: 10.1016/j.vetpar.2015.01.014 25665462

[B29] Li W. Liu X. Gu Y. Liu J. Luo J. (2019c). Prevalence of *Cryptosporidium*, Giardia, Blastocystis, and trichomonads in domestic cats in East China. J. Veterinary Med. Sci. 81, 890–896. doi: 10.1292/jvms.19-0111 PMC6612483 31105139

[B30] Li J. Ryan U. Guo Y. Feng Y. Xiao L. (2021). Advances in molecular epidemiology of cryptosporidiosis in dogs and cats. Int. J. Parasitol. 51, 787–795. doi: 10.1016/j.ijpara.2021.03.002 33848499

[B31] Li L. Sui Y. Li X. Song P. Chen G. Liu H.. (2024). Molecular characterization of *Cryptosporidium* spp. and *Giardia duodenalis* in pet cats in Henan Province, central China. Acta Tropica 254, 107188. doi: 10.1016/j.actatropica.2024.107188 38531428

[B32] Maritz J. M. Land K. M. Carlton J. M. Hirt R. P. (2014). What is the importance of zoonotic trichomonads for human health? Trends Parasitol. 30, 333–341. doi: 10.1016/j.pt.2014.05.005 24951156 PMC7106558

[B33] Meng X.-Z. Li M.-Y. Lyu C. Qin Y.-F. Zhao Z.-Y. Yang X.-B.. (2021). The global prevalence and risk factors of *Cryptosporidium* infection among cats during 1988–2021: A systematic review and meta-analysis. Microbial Pathogenesis 158, 105096. doi: 10.1016/j.micpath.2021.105096 34273476

[B34] Mohamed A. S. Glickman L. T. Camp J. W. Lund E. Moore G. E. (2013). Prevalence and risk factors for Giardia spp. infection in a large national sample of pet dogs visiting veterinary hospitals in the United States, (2003–2009). Veterinary Parasitol. 195, 35–41. doi: 10.1016/j.vetpar.2012.12.049 23337331

[B35] Morelli S. Diakou A. Di Cesare A. Colombo M. Traversa D. (2021). Canine and feline parasitology: Analogies, differences, and relevance for human health. Clin. Microbiol. Rev. 34, e00266–e00220. doi: 10.1128/CMR.00266-20 34378954 PMC8404700

[B36] Nagamori Y. Payton M. E. Looper E. Apple H. Johnson E. M. (2020). Retrospective survey of parasitism identified in feces of client-owned cats in North America from 2007 through 2018. Veterinary Parasitol. 277, 109008. doi: 10.1016/j.vetpar.2019.109008 31841945

[B37] Önder Z. Yetişmiş G. Pekmezci D. Delibaşı Kökçü N. Pekmezci GökmenZ. Çiloğlu A.. (2021). Investigation of zoonotic *Cryptosporidium* and *Giardia intestinalis* species and genotypes in cats (felis catus). Turkish J. Parasitol. 45, 252–256. doi: 10.4274/tpd.galenos.2021.46320 34889191

[B38] Pan W. Wang M. Abdullahi A. Y. Fu Y. Yan X. Yang F.. (2018). Prevalence and genotypes of *Giardia lamblia* from stray dogs and cats in Guangdong, China. Veterinary Parasitology: Regional Stud. Rep. 13, 30–34. doi: 10.1016/j.vprsr.2018.03.012 31014884

[B39] Pipiková J. Papajová I. Majláthová V. Šoltys J, Bystrianska J. Schusterová I.. (2020). First report on *Giardia duodenalis* assemblage F in Slovakian children living in poor environmental conditions. J. Microbiology Immunol. Infection 53, 148–156. doi: 10.1016/j.jmii.2018.04.007 29907537

[B40] Qiu L. Xia W. Li W. Ping J. Ding S. Liu H. (2019). The prevalence of microsporidia in China : A systematic review and meta-analysis. Sci. Rep. 9, 3174. doi: 10.1038/s41598-019-39290-3 30816168 PMC6395699

[B41] Romatowski J. (2000). *Pentatrichomonas hominis* infection in four kittens. J. Am. Veterinary Med. Assoc. 216, 1270–1272. doi: 10.2460/javma.2000.216.1270 10767968

[B42] Rommel M. (1985). *Sarcocystosis* of domestic animals and humans. In Pract. 7, 158–160. doi: 10.1136/inpract.7.5.158 3932221

[B43] Rosypal A. C. Ripley A. Stockdale Walden H. D. Blagburn B. L. Grant D. C. Lindsay D. S. (2012). Survival of a feline isolate of *Tritrichomonas foetus* in water, cat urine, cat food and cat litter. Veterinary Parasitol. 185, 279–281. doi: 10.1016/j.vetpar.2011.11.003 22100399

[B44] Ryu H. Alum A. Mena K. D. Abbaszadegan M. (2007). Assessment of the risk of infection by *Cryptosporidium* and *Giardia* in non-potable reclaimed water. Water Sci. Technol. 55, 283–290. doi: 10.2166/wst.2007.047 17305151

[B45] Santín M. Fayer R. (2011). Microsporidiosis: *Enterocytozoon bieneusi* in domesticated and wild animals. Res. Veterinary Sci. 90, 363–371. doi: 10.1016/j.rvsc.2010.07.014 20699192

[B46] Strazdaitė-Žielienė Ž. Baranauskaitė A. Butkauskas D. Servienė E. Prakas P. (2022). Molecular identification of parasitic protozoa *Sarcocystis* in water samples. Veterinary Sci. 9, 412. doi: 10.3390/vetsci9080412 PMC9412564 36006327

[B47] Turner D. C. (2017). A review of over three decades of research on cat-human and human-cat interactions and relationships. Behav. Processes 141, 297–304. doi: 10.1016/j.beproc.2017.01.008 28119016

[B48] Xenoulis P. G. Lopinski D. J. Read S. A. Suchodolski J. S. Steiner JörgM. (2013). Intestinal *Tritrichomonas foetus* infection in cats: a retrospective study of 104 cases. J. Feline Med. Surg. 15, 1098–1103. doi: 10.1177/1098612X13495024 23838083 PMC10816472

[B49] Xiao L. (2010). Molecular epidemiology of cryptosporidiosis: an update. Experimental Parasitology. 124 (1), 8. doi: 10.1016/j.exppara.2009.03.018 19358845

[B50] Xiao L. Fayer R. Ryan U. Upton S. J. (2004). *Cryptosporidium* taxonomy: recent advances and implications for public health. Clin. Microbiol. Rev. 17, 72–97. doi: 10.1128/CMR.17.1.72-97.2004 14726456 PMC321466

[B51] Xu H. Jin Y. Wu W. Li P. Wang L. Li N.. (2016). Genotypes of *Cryptosporidium* spp., *Enterocytozoon bieneusi* and *Giardia duodenalis* in dogs and cats in Shanghai, China. Parasites Vectors 9, 121. doi: 10.1186/s13071-016-1409-5 26932267 PMC4774012

[B52] Yao C. Köster L. S. (2015). *Tritrichomonas foetus* infection, a cause of chronic diarrhea in the domestic cat. Veterinary Res. 46, 35. doi: 10.1186/s13567-015-0169-0 PMC4364588 25880025

